# Supplementary material for: Ewing Sarcoma-Derived Extracellular Vesicles Impair Dendritic Cell Maturation and Function
Source: Cells. 2021 Aug 13;10(8):2081. doi: 10.3390/cells10082081 (PMC8391167; doi:10.3390/cells10082081)
Supplement: Supplementary file 1 [file cells-10-02081-s001.zip › cells-1308164-SI.pdf]

Supplementary Figures:

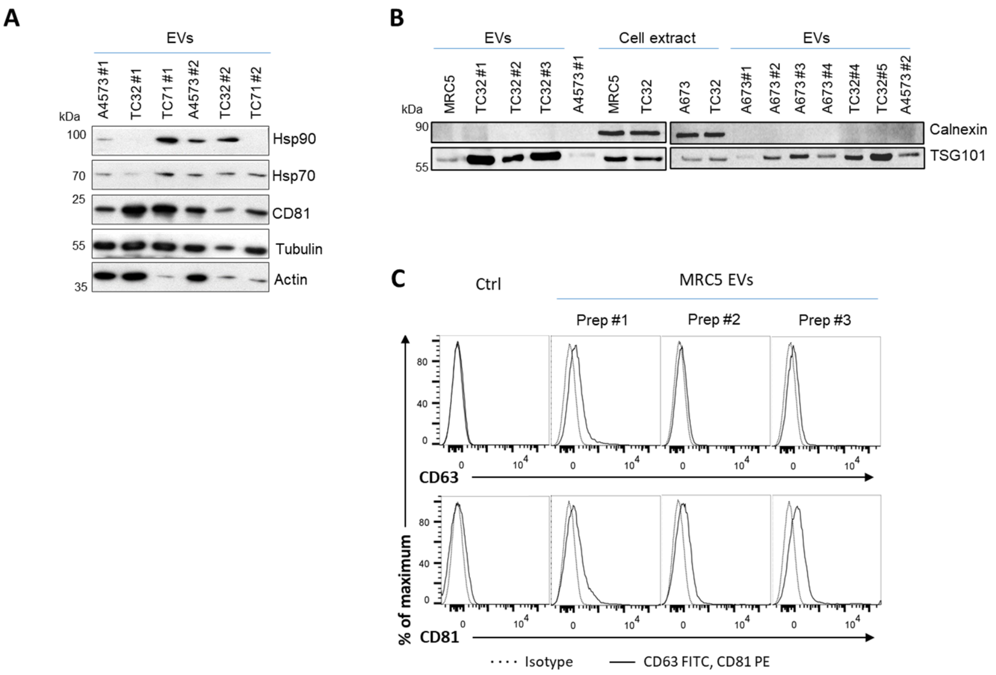

Supplementary Figure S1. Extended purification and characterization of EV preparations from EwS and fibroblast cell lines and healthy donor plasma. (**A**) Immunoblotting-detection of Hsp70 and Hsp90 chaperon proteins, CD81 EV marker, Tubulin and Actin in the purified EV preparations from EwS cell lines is shown. (**B**) Immunoblotting-detection of EV marker TSG101 and the negative marker Calnexin in the purified EV preparations and the respective parental cells. (**C**) Expression of the CD63 and CD81 EV markers (solid line) compared to IT antibodies (dotted line) on three independent MRC5 EV preparations bound to 3.9 µm latex beads. Results were obtained from indicated number of independent EV preparations.

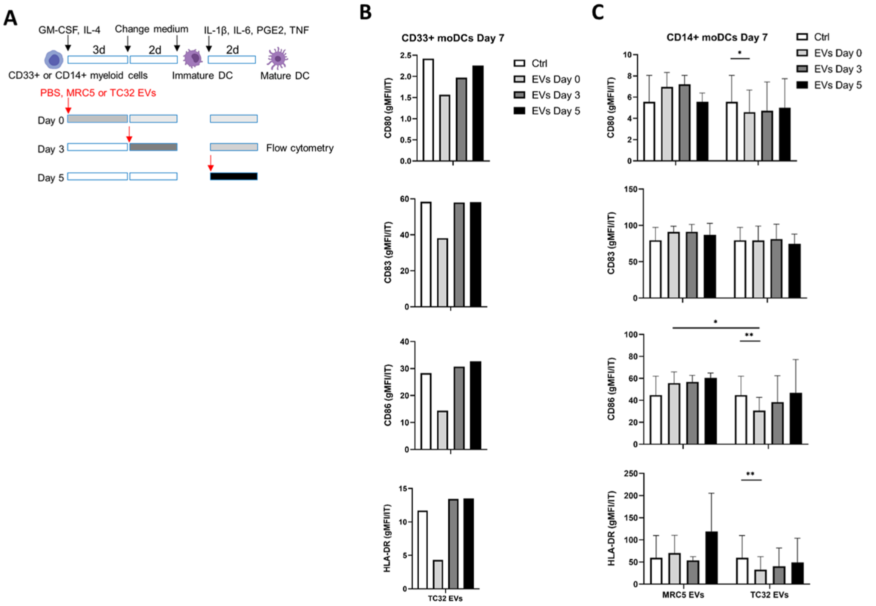

Supplementary Figure S2. Maturation of CD33⁺ and CD14⁺ myeloid cells is impaired by early exposure to EwS EVs. (**A**) Experimental design (B, C). CD33⁺ or CD14⁺ myeloid cells isolated from healthy donors were

differentiated with the GM-CSF and IL-4 cocktail for 5 days and matured with IL-1β, IL-6, PGE_2 and TNF for additional 2 days to moDCs. PBS (Control), MRC5 EVs or TC32 EVs (3 x 10^9/ml) were added either during differentiation (days 0 and 3) or maturation (day 5). Expression of maturation markers was assessed by flow cytometry at day 7. Geometric mean fluorescence intensity (gMFI) of CD80, CD83, CD86 and HLA-DR normalized to IT antibodies of $CD33^+$ myeloid cells (**B**) or $CD14^+$ monocytes (**C**). Results were obtained from one (**B**) or six (**C**) independent donors with one (**B**), three (**C**, MRC5 EVs) or five (**C,** TC32 EVs) independent EV preparations. Data are presented as mean ± SD. Paired t-test (B, C) was used to calculate P values. *p ≤ 0.05, **p ≤ 0.01.

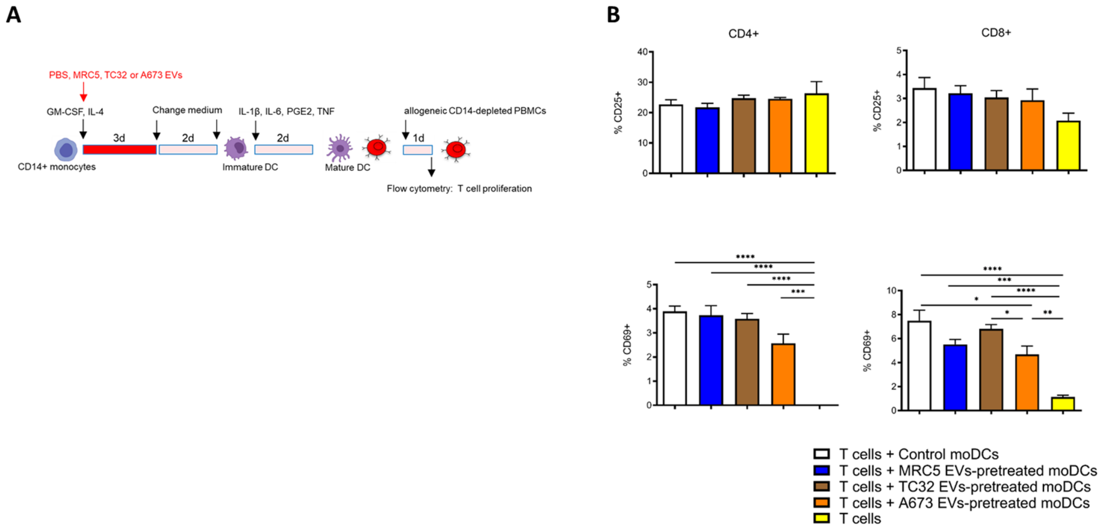

Supplementary Figure S3. $CD8^+$ T cells fail to upregulate the CD69 (but not CD25) activation marker when co-cultured with allogeneic $CD14^+$ monocytes differentiated in the presence of EwS EVs. Experimental design **(A)** and co-culture experiments (**B**). $CD14^+$ monocytes differentiated in the presence of PBS or 3 x 10^9 EVs/ml from MRC5, TC32 or A673 cells were co-cultured in a 1:2 ratio with allogeneic CD14-depleted PBMCs for 24 h. Percentage of CD25 and CD69 surface expression on $CD4^+$ and $CD8^+$ T cells was determined by flow cytometry. Results are representative of two independent donors and three independent EV preparations. Data are presented as mean ± SD. One-way ANOVA with multiple comparison Turkey test was used to calculate P values. *p ≤ 0.05, **p ≤ 0.01, ***p ≤ 0.001 and ****p ≤ 0.0001.

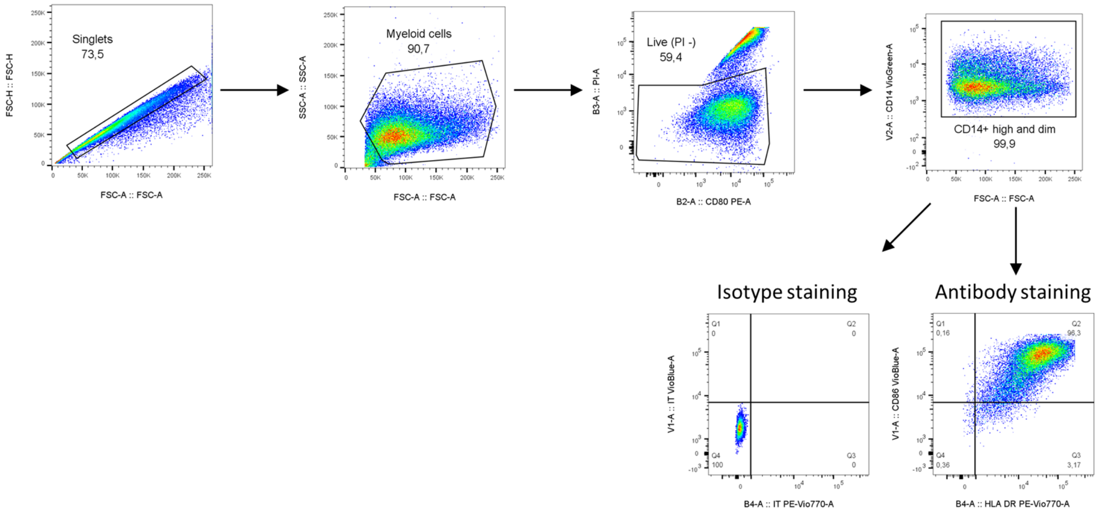

Supplementary Figure S4. Flow cytometry gating strategy of myeloid cells.
